# The national moroccan registry of ST-elevation myocardial infarction (MR-MI)

**DOI:** 10.1186/s12872-023-03458-7

**Published:** 2023-08-24

**Authors:** Aida Soufiani, Hamza Chraibi, Ilyasse Asfalou, Noha El Ouafi, Mustapha El Hattaoui, Rachida Habbal, Ali Chaib, Rokya Fellat, Hafid Akoudad, Aatif Benyass, Mohamed Cherti, Redouane Abouqal, Nesma Bendagha, Samir Ztot

**Affiliations:** 1 Cardiology A Department, Ibn Sina Hospital University Centre, Mohammed V University, Rabat, Morocco; 2 Cardiology Department, Mohammed V Military Instruction Hospital, Mohammed V University, Rabat, Morocco; 3 Cardiology Department, Mohammed VI Hospital University Centre, Mohamed Premier University, Oujda, Morocco; 4 Cardiology Department, Mohammed VI Hospital University Centre, Cadi Ayyad University, Marrakesh, Morocco; 5 Cardiology Department, Ibn Rochd Hospital University Centre, Hassan II University, Casablanca, Morocco; 6 Cardiology Department, Hassan II Hospital University Centre, Sidi Mohamed Ben Abdellah University, Fez, Morocco; 7 Cardiology B Department, Ibn Sina Hospital University Centre, Mohammed V University, Rabat, Morocco; 8 Medical Emergencies Department, Ibn Sina Hospital University Centre, Mohammed V University, Rabat, Morocco

**Keywords:** Acute coronary syndrome, Acute myocardial infarction, STEMI

## Abstract

**Background:**

MR-MI is the first national Moroccan ST-elevation myocardial infarction (STEMI) registry. Its objectives are to assess patient management modalities and highlight the clinical and therapeutic characteristics of this pathology in all cardiology centres on a national scale.

**Methods:**

Adult patients presenting with STEMI within 5 days of symptoms onset were enrolled over a period of 18 weeks from April to August 2018. 57 cardiology centres distributed in 22 cities in Morocco participated in the study, including 5 university hospitals, representing 70% of Moroccan centres managing STEMI patients. A case report form was sent to the investigators in both electronic and paper forms. Sociodemographic, clinical, management, revascularization, and follow-up data were collected.

**Results:**

A total of 809 patients were recruited. The population was mostly male (74.8%) with an average age of 62.6 ± 11.6 years. The most common risk factors were smoking (38.3%) arterial hypertension (30.7%), and diabetes (28%). 30% of patients were admitted within the first 6 h of symptoms onset and early revascularization was performed on 49.6%. Mortality rate was 5.2% in-hospital and 3.2% at the one-month follow-up.

**Conclusion:**

MR-MI is the first Moroccan STEMI registry on a national scale. Relevant management delays are much longer than other countries, and less than 50% of the patients that present on time benefit from early revascularization. Efforts remain to be done on the optimal diagnosis and treatment of STEMI.

## Background

Despite global improvements in management, cardiovascular disease, and more specifically ischemic heart disease (IHD) still represents a substantial health burden, with major social and economic consequences [1–4].

In Morocco, IHD has become the number one cause of mortality, with a 31% rate according to the 2019 Global Burden of Diseases Study [1]. Among cardiovascular risk factors, a recent meta-analysis remarked that tobacco smoking (20 to 45%) and hypertension (25 to 30%) are the most prevalent in the Moroccan population [5]. Management of ST-elevation myocardial infarction (STEMI) remains far from optimal; in a study published in 2012 regrouping patients from Morocco, Tunisia, and Algeria, Moustaghfir et al. reported that almost half of the patients do not receive any reperfusion therapy, explaining the higher rate of 30-day mortality compared to other countries (4.1%) [6].

The 2018 MR-MI (Moroccan Registry of Myocardial Infarction) is a project of the Moroccan Society of Cardiology in partnership with the National College of Myocardial Infarction. Our goals were:

- to extensively characterize the clinical profile of STEMI patients throughout Morocco’s regions, and set up a reference database for future studies;
- to quantitatively and qualitatively assess the current state of STEMI management in Morocco, and consequences on short- and long-term patient outcomes;
- to study the implementation of relevant practice guidelines in a real-world setting.

Large-scale descriptive studies are far and few in Morocco. The largest myocardial infarction registry is a city-wide effort from Fez, published in 2015 by Akoudad et al. and compiling 1835 patients [7]. To our knowledge, this is the first national scale STEMI registry in Morocco.

## Methods

### Study design

This was a national prospective multicentre study, including 70% of Moroccan centres managing STEMI patients.

### Study population

Data was collected over a period of 18 weeks, from April to August 2018.

Inclusion criteria were: patients aged > 18 years; admitted for recent (less than 5 days) STEMI, defined as ischaemic symptoms such as chest pain alongside an acute rise of troponin or another cardiac enzyme above the 99th percentile upper reference limit, and at least one of the following: ST-segment elevation in two or more contiguous leads, new abnormal Q waves or new-onset bundle branch block on admission electrocardiogram (ECG).

Exclusion criteria were: STEMI with symptom onset > 5 days; patients admitted for NSTE-ACS (non ST-elevation acute coronary syndrome), defined as chest pain and/or rise of troponin values without ST-segment elevation; patients admitted in cardiac arrest, who died in transport or very early after admission.

All patients gave informed consent to participate in the study.

### Study organization

#### Participating centres

**Fig. 1:**
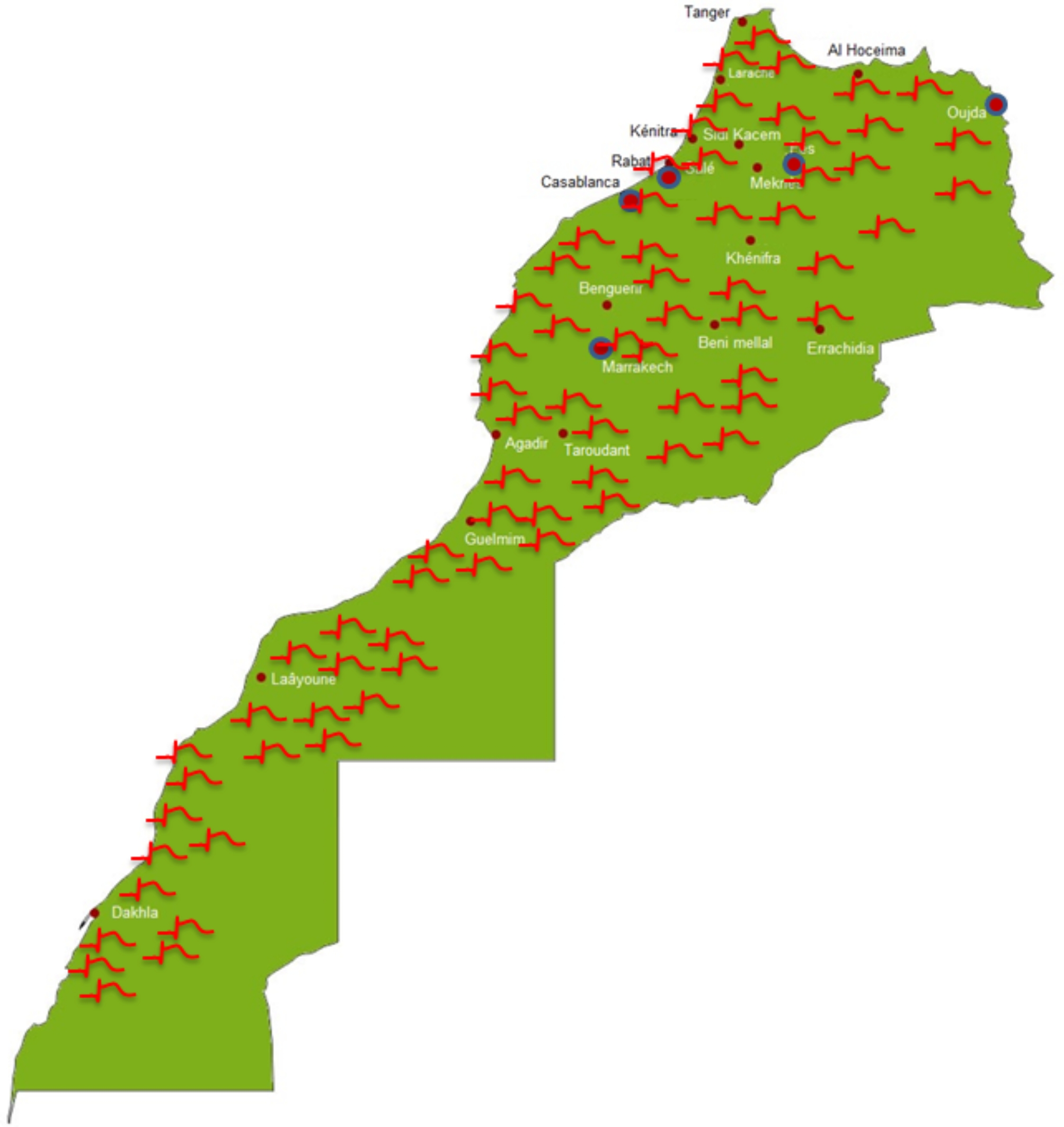
Geographical map of participating centres

57 centres across 22 cities accepted to participate in the study, including university and provincial hospitals, military hospitals, and private clinics, and covering a large majority of the Moroccan territory (Fig. 1). 5 university hospitals were represented (Rabat, Casablanca, Fez, Oujda, Marrakech).

#### Data collection

A standardized case report form was sent to all the participating centres and was filled by the onsite investigator or the attending physician. Sociodemographic data such as mode of living and health coverage was collected. Clinical data included medical history and cardiovascular risk factors, STEMI symptoms and timing, vital constants and Killip class at admission, findings on the first ECGand transthoracic echocardiogram (TTE) such as wall motion abnormalities and LVEF (left ventricular ejection fraction). TTE was performed by the attending physician using onsite available ultrasound machines. LVEF was calculated on the 2-chamber and 4-chamber apical views using Simpson’s biplane method. Wall motion abnormalities were defined as the presence of hypokinesia, akinesia or dyskinesia on one or more myocardial wall segments. The speed and quality of management was thoroughly documented; revascularization modalities, angiographic findings, and relevant time delays (symptoms-to-first medical contact and symptoms-to-management) were precisely recorded. For the purpose of symptoms-to-management time measurements, management was defined as either fibrinolysis injection or stent deployment in the context of percutaneous coronary intervention (PCI). Coronary angiography acquisition views and image interpretation were at the discretion of the attending interventional cardiologist. The culprit artery was determined by the presence of arterial occlusion or severe stenosis (> 70%) on a major epicardial artery, assuming it corresponded with the patient’s electrocardiographic and echocardiographic data. Follow-up data included in-hospital complication and mortality rates, and outcomes at one month.

#### Data quality

Data quality measures, such as manual and automated checks by the investigators, were undertaken throughout the study. All the data entered were verified and corrected by external assistants, and carefully stored in a protected electronic database. The amount of missing data was judged acceptable (less than 5% for all variables).

### Statistical analysis

Qualitative variables are reported as means ± standard deviations (SD), and median values were calculated when appropriate. Discrete variables are reported as percentages.

## Results

### Baseline characteristics

A total of 809 STEMI patients was included, originating from 76 cities and 68 rural villages. The majority were admitted in university hospitals (52.9%), followed by regional hospitals (20.7%) and private centres (14.3%). 15% didn’t have any health coverage (Table 1).

**Table 1:** Baseline characteristics of STEMI patients

Variables	Patients (N = 809)
*Centre*	
University hospital (%)	52.3
Regional hospital (%)	20.7
Military hospital (%)	10.8
Private clinic (%)	14.3
Private practice (%)	1.9
*Demographics*	
Age (years)	62.6 ± 11.6
Women (%)	25.2
Urban origin (%)	76.5
Rural origin (%)	23.5
Health coverage (%)	85.0
*Cardiovascular risk factors*	
Diabetes (%)	28.0
Arterial hypertension (%)	30.7
Smoking (%)	38.3
Dyslipidaemia (%)	13.6
Body mass index	26.6 ± 4.1
Family history of CAD (%)	7.5
*Cardiovascular history and comorbidities*	
Angina (%)	74.2
Myocardial infarction (%)	16.6
PCI (%)	8.6
CABG (%)	1.0
Stroke (%)	3.0
Peripheral artery disease (%)	2.6
Chronic kidney disease (%)	8.6

Data are expressed as percentage (%) or mean ± SD. CABG: coronary artery bypass graft; CAD: coronary artery disease; PCI: percutaneous coronary intervention; STEMI: ST-segment-elevation myocardial infarction.

The mean age was 62.6 ± 11.6 years, and 42% of patients were younger than 60. The majority were men (74.8%). The most common modifiable cardiovascular risk factors were smoking (38.3%) and arterial hypertension (30.7%). 65% of patients had 3 or more risk factors. 63% had no cardiovascular medical history (Table 2).

**Table 2:** Initial presentation

Variables	Patients (N = 809)
*First medical contact*	
Emergency department (%)	68.8
Private cardiology practice (%)	22.8
Private general practice (%)	8.4
EMS involvement (%)	3.2
*Initial symptoms*	
Typical chest pain (%)	56.4
Atypical chest pain (%)	6.1
Heart failure (%)	13.4
Syncope (%)	1.1
Cardiac arrest (%)	0.5
Others (%)	6.0
*Initial Killip class*	
I (%)	73.2
II (%)	20.2
III (%)	4.3
IV (%)	2.1
*Admission parameters*	
Heart rate (beats per minute)	85.6 ± 19.6
Systolic blood pressure (mmHg)	131.5 ± 26.4
Diastolic blood pressure (mmHg)	78.0 ± 16.7
*Electrocardiographic findings*	
Sinus rhythm (%)	80.4
Atrial fibrillation or flutter (%)	3.1
Ventricular arrythmia, including PVC (%)	4.9
ST-elevation (%)	63.8
Anterior leads (%)	43.3
Inferior leads (%)	32.5
Lateral leads (%)	11.9
Other leads (%)	2.6
Pathological Q waves (%)	32.4
LBBB or RBBB (%)	3.8
*Echocardiographic findings*	
TTE performed (%)	63.0
LVEF (%)	44.9 ± 10.8
Akinesia (%)	80.4
Hypokinesia (%)	88.2

Data are expressed as percentage (%) or mean ± SD. EMS: emergency medical services; LBBB: left bundle branch block; LVEF: left ventricular ejection fraction; PVC: premature ventricular contractions; LBBB: left bundle branch block; RBBB: right bundle branch block; TTE: transthoracic echocardiography.

The main complaint was typical chest pain (56.4%), followed by heart failure symptoms (13.4%). The majority were Killip I at admission (73.2%). The mean heart rate and blood pressures were 85 ± 20 beats per minute and 131 ± 26 / 78 ± 17 mmHg, respectively. Prehospital ECG was obtained only in 0.9% of cases. The most common arrhythmias were premature ventricular contractions (4.5%) and atrial fibrillation (AF) or flutter (3.1%). The majority of STEMI were anterior (47.9%) or inferior (36%). Mean LVEF was 45 ± 10%, and 27% of patients had a reduced LVEF (< 40%).

### Management and reperfusion

**Fig. 2:**
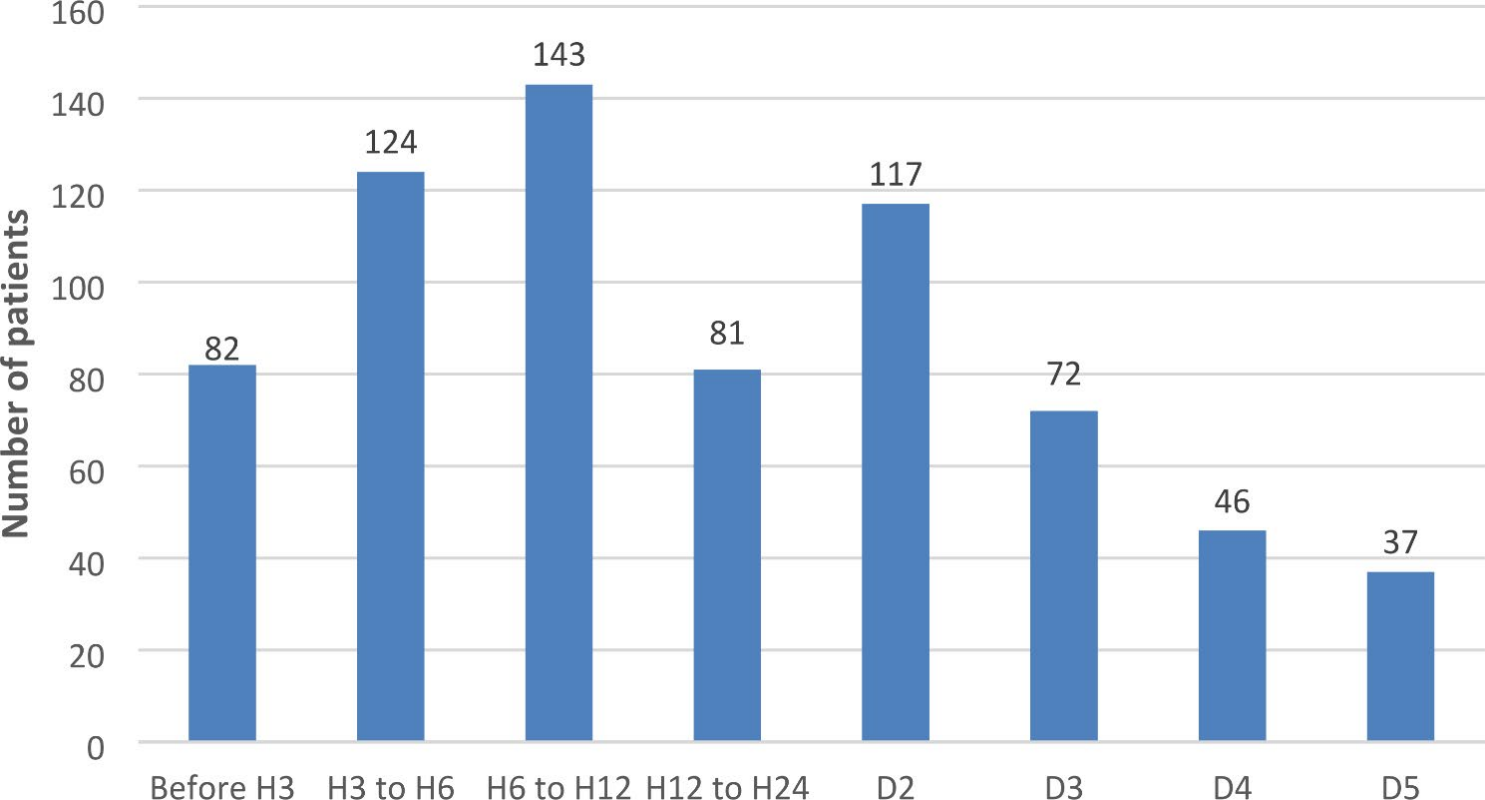
Symptoms to first medical contact delay

**Fig. 3:**
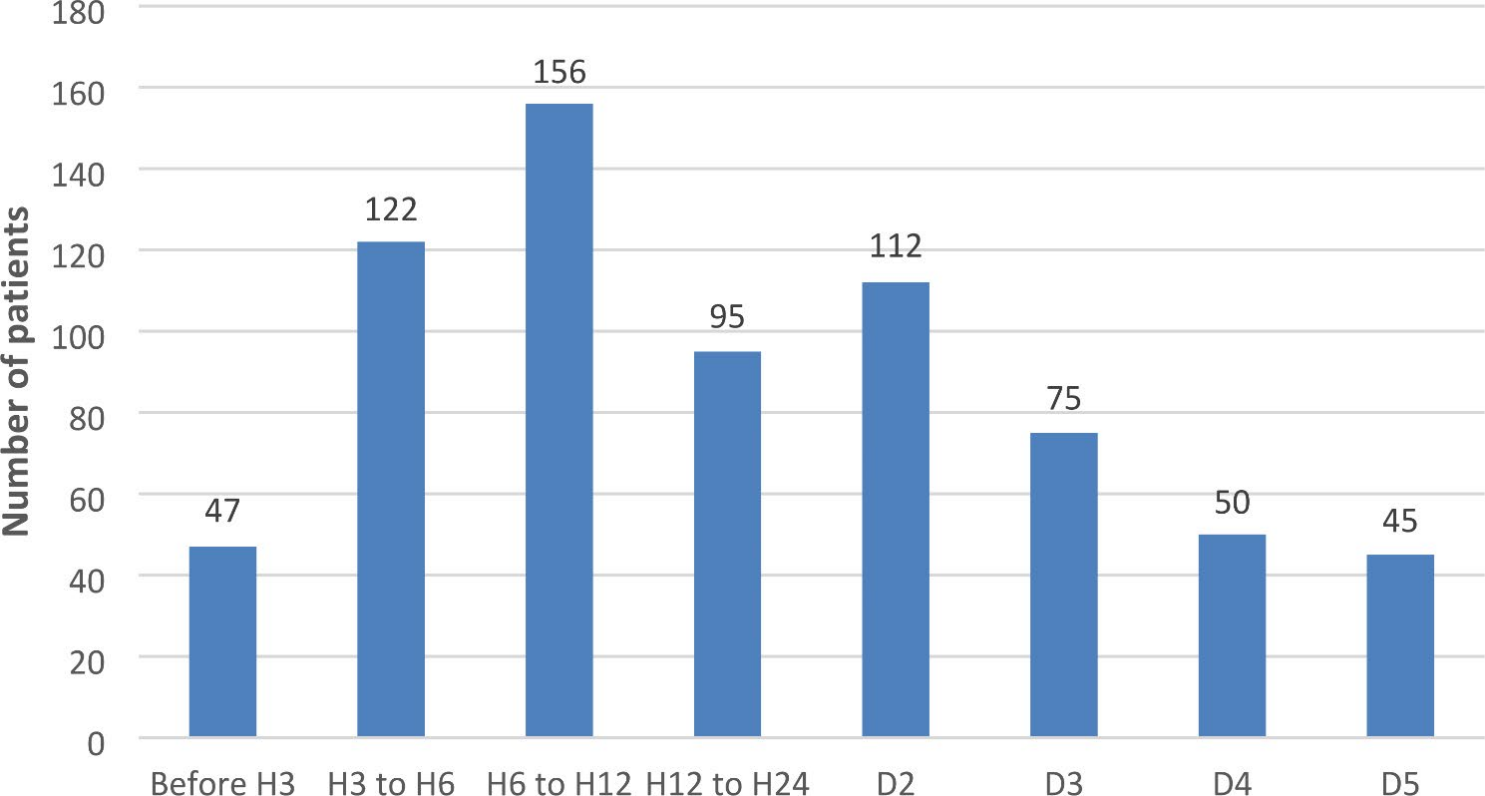
Symptoms to management delay

The median symptoms-to-first medical contact delay was 690 min and 50% of patients presented after the 12-hour mark (Fig. 2). The median symptoms-to-management delay was 815 min (Fig. 3). Regarding medical therapy, most patients received aspirin, clopidogrel and low molecular weight heparin. Fibrinolysis was performed in 23.4% of patients, with a median time delay of 6 h, with the majority being carried out in university hospitals. 98% of the 809 patients underwent coronary angiography, regardless of delay. 26.2% of patients underwent PCI, mostly in private centres and university hospitals, with a median delay of 3 h. The most common culprit artery, in 81.2% of cases, was the left anterior descending (Table 3).

**Table 3:** Initial management

Variables	Patients (N = 809)
*Procedures and revascularization*	
Coronary angiography (%)	98
Vascular access	
Radial (%)	53.7
Femoral (%)	46.1
Culprit artery	
Left anterior descending (%)	81.2
Circumflex (%)	10.9
Right coronary (%)	7.1
PCI (%)	26.2
PCI < 24 h from symptom onset (%)	14.3
Median delay (hours)	3
Drug-eluting stent (%)	21.5
Thrombus aspiration (%)	3.8
Fibrinolysis (%)	23.4
Median delay (hours)	6
*Medication*	
Aspirin (%)	80.3
Clopidogrel (%)	85.7
LMWH (%)	81.6
UFH (%)	9.3
Fondaparinux (%)	1.5
Glycoprotein IIb/IIIa inhibitor (%)	5.4

Data are expressed as percentage (%). LMWH: low molecular weight heparin; PCI: percutaneous coronary intervention; UFH: unfractionated heparin.

### Outcomes

In-hospital evolution was marked by a 17.4% rate of complications, the most prevalent being arrhythmias and recurrent ischemia. Mortality rate was 5.2%, with a mean age of 61 ± 11 years (Table 4). Follow-up data was available for 42.4% of the cohort. At the 1-month check, 94.1% of available patients were asymptomatic; the re-hospitalization (either for reinfarction or acute heart failure) and mortality rates were 2.1% and 3.8%, respectively (Table 5).

**Table 4:** In-hospital evolution and outcomes

Variables	Patients (N = 809)
*Complications (%)*	17.4
Ischemic recurrence (%)	1.7
Stent thrombosis (%)	0.6
Arrhythmia (%)	7.1
Conduction disturbance (%)	2.9
Mechanical complication (%)	4.9
*In-hospital mortality (%)*	5.2

Data are expressed as percentage (%).

**Table 5:** Follow-up results at one month

Variables	Patients (N = 343)
Asymptomatic (%)	94.1
Rehospitalization (%)	2.1
Mortality (%)	3.8
Data are expressed as percentage (%).	

## Discussion

MR-MI aimed to answer two important questions. First, what are the main differences between the Moroccan STEMI patients and those from other countries? Second, does the management in Morocco adhere to current practice guidelines? The size of our cohort (809 patients) was judged sufficient to answer these questions and is in line with other major registries such as Tunisia (459) [8] and France (1872 patients) [9] when adjusted to the population numbers.

To answer the first question, our results should be compared to other Mediterranean countries, such as Tunisia [8] and France [9]. Sociodemographic data is similar in all three countries, with a mean age of 60 to 63 years, and a clear male predominance (74.8% in Morocco and France, 81.5% in Tunisia). Smoking and arterial hypertension are the most common risk factors although hypercholesterolemia is more prevalent in France (36 vs. 13.6%) and smoking is much more frequent in Tunisia (63.6 vs. 38.3%). Smoking is a major health issue in Morocco; with a prevalence of 30 to 50% [5, 10]. That rate is probably higher due to the social stigma associated with smoking in Morocco, and the increase among young men of other forms of tobacco usage such as water pipe-smoking, which are not reported in many studies [5]. The prevalence of arterial hypertension in the Moroccan population ranges from 26 to 29% in most major reports [11–13]. Arterial hypertension is also severely underdiagnosed and undertreated in Morocco; in the ETHNA study, 29% of patients were newly diagnosed, and in treated hypertensive patients, control rates range from 25 to 35% [13, 14]. Hypercholesterolemia was probably underreported in our study as 29% of Moroccan patients suffer from this condition [13]. This discrepancy is probably explained by the fact that practicians are less likely to perform lipid panels in the emergency setting. In our study, 63% of patients had no cardiovascular medical history, which is lower than in France (84%). Typical chest pain was the most common presentation in all three countries, but heart failure was more frequent in Maghreb countries (13.4 and 11.4%) compared to France (3%), underlining the late presentation of patients in those countries. The prevalence of AF in our study was similar to the French one (3.1 vs. 4%). Anterior and inferior STEMI comprised about 90% of all localizations in the three countries. The mean LVEF was slightly increased in France compared to Morocco (50.2 vs. 45%). Overall, the clinical profile of STEMI patients remains similar between the three countries.

To answer the second question, it is essential to review revascularization modalities used and the various management delays. The 2017 European guidelines on STEMI recommend that a patient undergoes primary PCI within 48 h (but preferably 12 h) after symptom onset. Fibrinolysis should be administered within 12 h after symptom onset if PCI is not available within 2 h [15]. Early presentation of the patient is therefore crucial for optimal diagnosis and management. In Morocco, the symptom-to-first medical contact delay is much longer than in France (690 vs. 141 min). Half of the patients consult after the key 12-hour mark, which explains why two thirds of the patients do not receive thrombolytic agents even when they’re available. Fibrinolysis was used in 23.4% of cases; that rate is higher in Tunisia (31.8%) but much lower in France (6%), underlining the poor availability of cath labs especially in rural areas and provincial hospitals. Only 14.3% benefit from primary PCI, compared to 30% in the Tunisian cohort and 91% in the French registry. These longer delays explain in part the higher rate of complications in Moroccan patients compared to French patients, especially recurrent myocardial infarction (1.7 vs. 0.3%) and in-hospitality death (5.2% vs. 2.8%). The mortality rate reported in our study is slightly inferior to the Tunisian one (5.2 vs. 5.5%). At the one-month follow-up, it remains stable at 3.8%.

What are concrete solutions to improve the situation in Morocco? We need to take a look at the 1995 to 2010 period in France, when 30-day mortality fell from 13.7 to 4.4% [16]. In addition to the STEMI demographic shift (patients were younger by 3 years on average), this decrease was mostly explained by shorter management delays (120 to 74 min), a greater involvement of emergency medical services (EMS), whose rate of use grew from 23 to 49% and a higher rate of primary angioplasty (49 to 75%) [17]. EMS involvement rate was only 3.2% in our study. By increasing the awareness of the general public about STEMI and the need to call EMS as soon as symptoms begin, management delays would be tremendously reduced, allowing for more efficient revascularization and better outcomes. The availability of cath labs is also an issue, especially in provincial hospitals where the only recourse is fibrinolysis, which is known to be inferior to PCI [7, 18]. These are the main areas that Morocco is developing in the present and the near future. Since 2018, the Moroccan government has made great strides in the construction of new PCI centres, both private and public, and the development of chest pain awareness programs.

## Conclusions

MR-MI is the first national STEMI registry in Morocco with a total of 809 patients and draws some important conclusions. The clinical profile of our patients is similar to that of other Mediterranean countries, but management suffers from many inadequacies, especially long time delays and inefficient revascularization options.

Concrete, proven solutions exist, such as the involvement of EMS and the democratization of urgent angiography and PCI. Since 2018, Moroccan practicians and health officials have been involved in the development of these areas and much progress has been made in the right direction.

## Data Availability

The datasets used and/or analyzed in this study are available from the corresponding author on reasonable request.

## Abbreviations

ECG: electrocardiogram
EMS: emergency medical services
IHD: ischemic heart disease
LVEF: left ventricular ejection fraction
PCI: percutaneous coronary intervention
STEMI: ST-elevation myocardial infarction
TTE: transthoracic echocardiogram

## References

[1] Roth GA, Mensah GA, Johnson CO, Addolorato G, Ammirati E, Baddour LM, GBD-NHLBI-JACC Global Burden of Cardiovascular Diseases Writing Group. ;. Global Burden of Cardiovascular Diseases and Risk Factors, 1990–2019: Update From the GBD 2019 Study. J Am Coll Cardiol. 2020;76(25):2982–3021. Erratum in: J Am Coll Cardiol. 2021;77(15):1958–1959. 10.1016/j.jacc.2020.11.010 PMC7755038 33309175

[2] Townsend N, Kazakiewicz D, Lucy Wright F, Timmis A, Huculeci R, Torbica A, Gale CP, Achenbach S, Weidinger F, Vardas P (2022). Epidemiology of cardiovascular disease in Europe. Nat Rev Cardiol.

[3] Bauersachs R, Zeymer U, Brière JB, Marre C, Bowrin K, Huelsebeck M (2019). Burden of Coronary Artery Disease and Peripheral Artery Disease: A literature review. Cardiovasc Ther.

[4] Keates A, Mocumbi A, Ntsekhe M, Sliwa K, Stewart S (2017). Cardiovascular disease in Africa: epidemiological profile and challenges. Nat Rev Cardiol.

[5] Elyamani R, Soulaymani A, Hami H (2021). Epidemiology of Cardiovascular Diseases in Morocco: a systematic review. Rev Diabet Stud.

[6] Moustaghfir A, Haddak M, Mechmeche R (2012). Management of acute coronary syndromes in Maghreb countries: the access (acute coronary events – a multinational survey of current management strategies) registry. Arch Cardiovasc Dis.

[7] Akoudad H, El Khorb N, Sekkali N, Mechrafi A, Zakari N, Ouaha L et al. L’infarctus du Myocarde au Maroc: Les Données du Registre Fes-Ami. Annales de Cardiologie et d’Angéiologie. 2015;64(6):434–8. 10.1016/j.ancard.2015.09.050 26492984

[8] Addad F, Mahdhaoui A, Gouider J, Boughzela E, Kamoun S, Boujnah MR et al. Management of patients with acute ST-elevation myocardial infarction: results of the fast-mi tunisia registry. PLoS ONE. 2019;14(2). 10.1371/journal.pone.0207979 PMC6386252 30794566

[9] Belle L, Cayla G, Cottin Y, Coste P, Khalife K, Labèque J-N (2017). French registry on Acute ST-elevation and non – ST-elevation myocardial infarction 2015 (FAST-MI 2015). Design and baseline data. Arch Cardiovasc Dis.

[10] Nejjari C, Benjelloun MC, Berraho M, El Rhazi K, Tachfouti N, Elfakir S (2009). Prevalence and demographic factors of smoking in Morocco. Int J Public Health.

[11] Tazi MA, Abir-Khalil S, Chaouki N, Cherqaoui S, Lahmouz F, Sraïri JE (2003). Prevalence of the main cardiovascular risk factors in Morocco: results of a National Survey, 2000. J Hypertens.

[12] Ministry of Health of Morocco. National survey of noncommunicable diseases risk factors 2017–2018. https://www.sante.gov.ma/Documents/2019/05/Rapport%20de%20l%20enqu%C3%AAte%20Stepwise.pdf.

[13] Nejjari C, Arharbi M, Chentir MT, Boujnah R, Kemmou O, Megdiche H (2013). Epidemiological trial of hypertension in North Africa (ETHNA): an international multicentre study in Algeria, Morocco and Tunisia. J Hypertens.

[14] Essayagh T, Essayagh M, El Rhaffouli A, Khouchoua M, Bukassa Kazadi G, Khattabi A (2019). Prevalence of uncontrolled blood pressure in Meknes, Morocco, and its associated risk factors in 2017. PLoS ONE.

[15] Ibanez B, James S, Agewall S, Antunes MJ, Bucciarelli-Ducci C, Bueno H (2018). 2017 ESC Guidelines for the management of acute myocardial infarction in patients presenting with ST-segment elevation: the Task Force for the management of acute myocardial infarction in patients presenting with ST-segment elevation of the European Society of Cardiology (ESC). Eur Heart J.

[16] Puymirat E, Simon T, Cayla G, Cottin Y, Elbaz M, Coste P, Lemesle G, Motreff P, Popovic B, Khalife K, Labèque JN, Perret T, Le Ray C, Orion L, Jouve B, Blanchard D, Peycher P, Silvain J, Steg PG, Goldstein P, Guéret P, Belle L, Aissaoui N, Ferrières J, Schiele F, Danchin N, USIK (2017). USIC 2000, and FAST-MI investigators. Acute myocardial infarction: changes in patient characteristics, management, and 6-Month Outcomes over a period of 20 years in the FAST-MI program (French Registry of Acute ST-Elevation or Non-ST-Elevation myocardial infarction) 1995 to 2015. Circulation.

[17] Puymirat E, Simon T, Steg PG, Schiele F, Guéret P, Blanchard D, USIK USIC 2000 Investigators; FAST MI Investigators. ;. Association of changes in clinical characteristics and management with improvement in survival among patients with ST-elevation myocardial infarction. JAMA 2012; 308:998–1006. 10.1001/2012.jama.11348 PMID: 22928184. 10.1001/2012.jama.11348 22928184

[18] Widimsky P (2010). Primary angioplasty vs. thrombolysis: the end of the controversy?. Eur Heart J.

